# Protective Effects of Ellagic Acid Against Arsenic‐Induced Hepatotoxicity in Male Wistar Rats: Impact of Heme Oxygenase‐1 Upregulation

**DOI:** 10.1002/fsn3.70344

**Published:** 2025-05-23

**Authors:** Turki Alkully, Mohamed F. El‐Refaei, Eman A. A. Abdallah

**Affiliations:** ^1^ Faculty of Medicine Al‐Baha University Al‐Baha Saudi Arabia; ^2^ Genetic Institute Sadat City University Sadat City Egypt; ^3^ Faculty of Medicine Zagazig University Zagazig Egypt

**Keywords:** arsenic trioxide, ellagic acid, HO‐1, liver toxicity, vascular endothelial growth factor

## Abstract

Hepatotoxins, which may originate from chemicals, prescription medications, or medicinal plants, are a major cause of hepatotoxicity. This study aims to evaluate the protective role of ellagic acid (EA) in attenuating As^III^‐induced hepatotoxicity in male rats. Forty male rats were used and divided into four groups (10 each). Group I: Negative control. Group II received 60 mg/kg/day of EA. Group III was intoxicated with As^III^ at a dose of 10 mg/kg body weight for 14 days. Group IV was intoxicated with As^III^ and treated daily with 60 mg/kg/day of EA, starting from Day 1 concurrently with As^III^ for a total of 14 days. According to the study, As^III^ intoxication (Group III) led to liver damage, as indicated by increased hepatocyte vascular endothelial growth factor (VEGF), heme oxygenase‐1 (HO‐1) expression, and elevated serum biochemical markers—alanine aminotransferase, aspartate aminotransferase, and alkaline phosphatase. Hepatocyte damage was confirmed through histopathological examination. In contrast, Group IV demonstrated reduced liver damage, mild VEGF expression, HO‐1 upregulation, and improvements in biochemical markers. Overall, the findings suggest that EA alleviated As^III^‐induced hepatotoxicity via the HO‐1 upregulation pathway, highlighting the therapeutic potential of EA in managing liver toxicity.

## Graphical abstract

Arsenic (As), a widespread environmental toxic substance, one of arsenic's clinical signs is liver damage. Ellagic acid protects liver toxicity as well as injuries induced by arsenic, and up‐regulates HO‐1 expression. Ellagic acid has a pharmacological characteristic, including anti‐inflammatory and antioxidant properties.

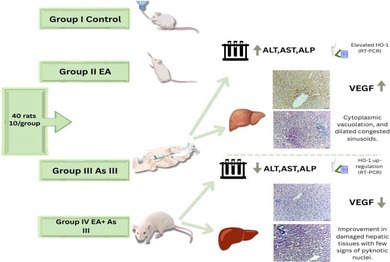

## 1 Introduction

The liver is exposed to innumerable exogenous substances that may lead to liver injury due to an immune system response toward these substances (McGill 2023). The damage incurred during the liver's inflammatory response to various chemicals is referred to as liver toxicity. Cirrhosis, liver fibrosis, and chronic liver disease are among the common outcomes of liver toxicity (Yu et al. 2017).

Medicinal substances, food, herbal supplements, and trace elements can all contribute to liver damage. Drug‐induced liver injury is the most frequent cause of liver toxicity. Biochemical indicators of liver disease may increase as a consequence of such toxicity. Hepatocellular injury, cholestatic injury, and mixed injury are the three main categories of abnormal biochemical changes in the liver (Cano et al. 2017; Hafeez et al. 2024).

Arsenic is a naturally occurring metalloid found in a wide variety of organic and inorganic forms. It is primarily present as trivalent arsenic (As^3+^) and pentavalent arsenic (As^5+^). Although compounds containing arsenic are toxic, trivalent arsenic is more harmful than the pentavalent form. Long‐term exposure to arsenic through contaminated water can lead to liver damage (Bashir et al. 2024). In animal models, chronic arsenic exposure has been associated with liver fibrosis and hepatocellular carcinoma (He et al. 2021; Liu and Waalkes 2008; Qian et al. 2023).

Ellagic acid (EA) is a naturally occurring secondary metabolite of bioactive polyphenolic compounds present in many different plant taxa. The pomegranate ( *Punica granatum* L.), as well as the wood and bark of certain tree species, contains a substantial amount of EA. Because of its anti‐inflammatory, anti‐mutagenic, antioxidant, and antiproliferative qualities, EA is becoming increasingly popular. The pharmacological effects of EA have been demonstrated in a range of in vitro and in vivo model systems. Moreover, the hepatoprotective, nephroprotective, and neuroprotective properties of EA have also been thoroughly investigated (Harper 2023; Xiao et al. 2022).

Ellagic acid's molecular actions include scavenging free radicals, controlling lipid metabolism, blocking fibrogenesis response‐mediating proteins, inhibiting hepatic stellate cells and myofibroblasts, limiting hepatic viral replication, facilitating growth factor suppression, controlling transcription factors and proinflammatory cytokines, enhancing the liver immune response, promoting apoptosis, and preventing the proliferation of tumorigenic cells (Aishwarya et al. 2021).

The present study aimed to investigate liver function tests, including biochemical parameters, to assess the protective properties of EA against toxicity induced by As^III^. Moreover, RT‐PCR was applied to estimate and examine heme oxygenase‐1 (HO‐1) expression and its role in redeeming hepatic cell injury in vivo. Furthermore, the histopathology of the liver for each group of rats will be taken into consideration to figure out and explicate the mode of action of EA and the impact of its protective properties in detail.

## 2 Materials and Methods

### 2.1 Ethics Statement

The animals were handled in accordance with the institution's policies and procedures for the use and care of laboratory animals, as well as the national guidelines published by the National Institutes of Health, an agency of the United States government (National Research Council 2011). The Committee on the Ethics of Animal Experiments, under the Ethics Committee Board at Al‐Baha University, Kingdom of Saudi Arabia, approved the protocol (REC/MED/BU‐FM/2024/1). In accordance with the American Veterinary Medical Association Guidelines, the euthanasia procedures form the basis of our Ethics Committee's legal and ethical standards. All surgeries were performed under sodium pentobarbital anesthesia, and every effort was made to minimize pain.

### 2.2 Animals

All procedures were carried out in compliance with the relevant rules and regulations. Forty adult, male Wistar rats, aged over 6 weeks, were provided by King Abdulaziz University, Jeddah, Saudi Arabia. The rats were housed for 5 days in plastic cages containing wood chip bedding, shredded paper, and a plastic enrichment tube at the animal house of the Biochemistry Department, Faculty of Medicine, Al‐Baha University, Saudi Arabia. Rats were kept in pairs and maintained on a 12‐h light/dark cycle (lights on at 7:00) in a controlled environment with a temperature of 24°C and humidity of 22%. The animals were pathogen‐free and tested negative for all antigens at the adjusted temperature. Feed was provided by Nutrition International, St. Louis, MO, USA, and water was available ad libitum.

### 2.3 Chemicals

#### 2.3.1 Arsenic Trioxide

The arsenic trioxide (As_2_O_3_; Sigma, St. Louis, MO, USA) (CAS No. 1327‐53‐3) was dissolved in water at a concentration of 10 mg/kg b.wt. Toxicity was achieved after 14 days of intraperitoneal (i.p.) administration. The biochemical and histopathological analyses carried out during the initial study confirmed the induction of liver toxicity in the rat groups. Using a 50% mortality rate over 15 days following i.p. administration of various doses (5, 10, 20, 40, 60, 80, and 100 mg/kg), the LD_50_ of As^III^ was determined. The percentage of rats that died was calculated against time and dose, using a plot of rats lost per Group versus the log of dosage to determine LD_50_ (Biram et al. 2023).

#### 2.3.2 Ellagic Acid

Ellagic acid (CAS No. 476–66‐4) was obtained from Sigma‐Aldrich Co. The effective dose was 60 mg/kg/day body weight for 14 days (Devipriya et al. 2007, 2008), dissolved in 0.1% dimethyl sulfoxide, and administered once daily via intragastric tube.

### 2.4 Experimental Design

At the start of the experiment, rats were divided into four groups (10 each). Group I was kept as the negative control. Group II received EA at a dose of 60 mg/kg per day. Group III was intoxicated with As^III^ at a dose of 10 mg/kg/b.wt (Patlolla et al. 2012) for 14 days (Celik et al. 2014; Mehrzadi et al. 2018) and received no treatment. Group IV was intoxicated with As^III^ and treated daily with EA at 60 mg/kg/b.wt, starting from day 1, concurrently with As^III^, for a total of 14 days (Table 1).

**TABLE 1:** Study design and drug administration.

Groups	No. of rats	Handling
I (Negative control)	10	Feed (Nutrition International, St. Louis, MO, USA, and water ad libitum were applied)
II/Treated EA	10	60 mg/kg/b.wt of Ellagic acid per day
III/Intoxicated As^III^	10	10 mg/kg/b.wt of As^III^ for 14 days
IV/Intoxicated As^III^ and EA	10	10 mg/kg/b.wt of As^III^ + 60 mg/kg/b.wt of Ellagic acid per day

After 24 h of the final treatment, the rats were anesthetized using ketamine hydrochloride (80 mg/kg, i.p.) and xylazine hydrochloride (10 mg/kg, i.p.). Blood samples were collected from each rat via heart puncture, and serum was stored for biochemical marker analysis. The liver was excised, cleaned, and prepared for HO‐1 RT‐PCR analysis. Additionally, the remaining liver tissue was preserved in 10% formalin for histopathological and immunohistochemical analyses. No rats died during the 15‐day experiment, and no signs of sores or irritability were observed in any of the rat groups.

### 2.5 Biochemical Analysis (Determination of Liver Function in the Serum of Different Rat Groups)

Under anesthesia, blood samples were collected from each rat, and the sera were separated by centrifugation at 3500 rpm for 10 min. The obtained sera were stored at −20°C until analysis. An autoanalyzer (Cobas 6000, Roche, Switzerland) was used to measure serum levels of alanine aminotransferase (ALT), aspartate aminotransferase (AST), and alkaline phosphatase (ALP) to assess the extent of hepatocellular damage (Makena et al. 2018). Serum samples from different rat groups were also analyzed for VEGF levels using the VEGF ELISA Kit (BEK‐2110‐1P, BEK‐2110‐2P) in accordance with the manufacturer's instructions (Laidding et al. 2021).

### 2.6 RT‐PCR for HO‐1 Expression

1 mL of TRIzol was used to homogenize 50 mg of the tissue. Following the manufacturer's instructions, RNA extraction was carried out (Invitrogen, Cat No. 15596026). For every 1 mL of TRIzol used, 0.2 mL of chloroform was added. The tubes were hand‐shaken vigorously for 15 s after being securely capped. The samples were then incubated at room temperature for 3 min. After incubation, the samples were centrifuged at 4000 rpm for 15 min at 4°C. The sample separated into a pale green organic phase, an interphase, and a colorless upper aqueous phase. The aqueous phase was removed for RNA extraction. With extreme caution, it was transferred to a different tube without disturbing the interphase.

To the aqueous phase, 0.5 mL of isopropyl alcohol was added. After 10 min of incubation at room temperature, the samples were centrifuged for 10 min at 4°C at 5000 rpm. The supernatant was removed, and the pellet was washed once with 75% ethanol, with at least 1 mL of ethanol added for every 1 mL of TRIzol used. The samples were vortexed and centrifuged for 5 min at 4°C at 3000 rpm.

Note: Samples can be stored for a week at 4°C or for a year at −20°C. After allowing the pellet to air dry, the solution was pipetted up and down to dissolve it in nuclease‐free water (Safwat et al. 2023). The RNA was stored at −70°C.

The Thermo Scientific NanoDrop One Microvolume UV–Vis Spectrophotometer was used to measure the concentration and purity of RNA. The RevertAid First Strand cDNA Synthesis Kit was used to reverse transcribe four μg of RNA into cDNA. The SensiFAST SYBR High‐ROX Kit, along with the StepOne Real‐Time PCR System (Applied Biosystems), was used to detect gene expression. The expression level was calculated using the 2–(ΔΔCt) method, where Ct is the cycle threshold. HO‐1 expression was evaluated by real‐time PCR using the forward and reverse primers, as described by Safwat et al. (2023) and Yin et al. (2021).

Sequences of primers for RT‐PCR.

**Table:** 

Gene	Primer's sequence
HO‐1	F: 5′‐TGCTTGTTTCGCTCTATCTCC‐3′
	R: 5′‐CTTTCAGAAGGGTCAGGTGTC‐3′
GAPDH	F: 5′‐TATCGGACGCCTGGTTAC‐3′
	R: 5′‐CTGTGCCGTTGAACTTGC‐3′

### 2.7 Immunohistochemical Examination of VEGF

In the present study, appropriate immunohistochemical analysis was used to determine VEGF expression in the experimental groups. From the paraffin‐embedded and formalin‐fixed tissue blocks, 4 μm‐thick liver sections were cut, deparaffinized, and rehydrated in ethanol for immunohistochemical staining. The sections were treated with normal horse serum for 30 min at room temperature and then with methanol containing 0.3% hydrogen peroxide for another 30 min. They were subsequently incubated overnight at 4°C with a 1:100 dilution of anti‐VEGF polyclonal antibody (Sigma‐Aldrich). As recommended by the manufacturer (Sigma‐Aldrich), a commercial kit was used to detect bound antibodies using the avidin–biotin‐labeled peroxidase complex method. Hematoxylin was used as the counterstain, and 3,3′‐diaminobenzidine tetrahydrochloride served as the chromogen (Cericl et al. 2020).

### 2.8 Histopathological Examination of the Liver

The liver was paraffin‐fixed, dried, and then placed in 10% paraformaldehyde before being sectioned at 5 μm thickness. Hematoxylin and eosin were used to stain the sections. Morphological alterations were examined under a microscope (Nikon Eclipse 80i, Japan), and images were captured using a Nikon DS‐Fi1 digital microscope camera (Bano and Najam 2019; Mohammad et al. 2023).

## 3 Statistical Analysis

A one‐way analysis of variance (ANOVA) test was used to compare the different groups under examination (more than two groups), and the data collected for each group were presented as mean ± standard deviation (SD). Significant differences between groups were further identified using the Tukey test. All computations were performed using an IBM computer and SPSS software, version 20. *p*‐values below 0.001 were considered highly significant, and *p*‐values below 0.05 were considered statistically significant.

## 4 Results

### 4.1 Biochemical Analysis

Group III displayed a substantial increase in serum biochemical markers—ALT, AST, and ALP levels (94.92 ± 2.22, 101.67 ± 1.12, and 125.91 ± 1.20 U/L, respectively)—compared with Group I. Increased blood levels of these enzymes signify hepatotoxicity, which is characterized by damage to liver cells due to As^III^ toxicity. Moreover, it may be a feature of oxidative damage through the interaction of As^III^ with the mitochondrial membrane, leading to loss of membrane permeability and generation of reactive oxygen species (ROS). This process, which results in structural and functional abnormalities in liver tissues, may help explain the pathophysiology behind liver function disturbances.

On the other hand, EA treatment (Group IV) exhibited a significant improvement in serum biochemical markers—ALT, AST, and ALP levels were 33.46 ± 1.25, 38.57 ± 0.52, and 67.50 ± 0.60 U/L, respectively (*p* < 0.001)—compared with Group III. This improvement in liver enzyme profiles is suspected to be due to the antioxidant, anti‐inflammatory, and anti‐fibrotic actions of EA.

Furthermore, there was a statistically significant (*p* < 0.05) increase in VEGF levels in the serum of rats intoxicated with As^III^, with an average level of 231.91 pg/mL on Day 15 in Group III. These results suggest that arsenic induces ROS, which in turn increase the expression of VEGF and other molecules. This activates signaling pathways and results in a proangiogenic microenvironment that promotes the development of liver damage. However, rats treated with EA (Group IV) showed improvements in serum VEGF levels, which were relatively reduced to 97.5 pg/mL (Table 2). The effect of EA might be attributed to a reduction in MMP activity, which could also be responsible for decreasing VEGF expression and thereby halting the neoangiogenesis process. EA may thus imply anti‐angiogenic properties.

**TABLE 2:** Mean ± SEM of serum ALT, AST, and ALP (U/L) and VEGF (pg/mL) levels in different groups of rats (*N* = 10).

	Control group I *N* = 10	Control negative treated EA group II *N* = 10	Intoxicated AsIII group III *N* = 10	Intoxicated AsIII treated EA group IV *N* = 10
ALT (U/l)	26.51 ± 0.95	28.91 ± 3.94	94.92 ± 2.22* ^,^ **	33.46 ± 1.25* ^,^ ** ^,^ ***
AST (U/l)	32.15 ± 0.56	32.59 ± 0.34	101.67 ± 1.12* ^,^ **	38.57 ± 0.52* ^,^ ** ^,^ ***
ALP (U/l)	58.81 ± 0.37	59.49 ± 0.99	125.91 ± 1.20* ^,^ **	67.50 ± 0.60* ^,^ ** ^,^ ***
VEGF (pg/ml)	56.81 ± 15.7	58.49 ± 16.1	231.91 ± 25.4* ^,^ **	97.5 ± 17.2* ^,^ ** ^,^ ***

*Note:* Data expressed as mean ± SD. Test used: One way ANOVA followed by post hoc tukey. *p* < 0.05; *p* < 0.01 versus Control negative group I. *p* < 0.05; *p* < 0.01 versus Control negative treated EA group II. *p* < 0.05; *p* < 0.01 versus Intoxicated As^III^ group III.

Abbreviations: P, probability; SD, standard deviation.

* *p* < 0.001 versus Control negative group I.

** *p* < 0.001 versus Control negative treated EA group II.

*** *p* < 0.001 versus Intoxicated As^III^ group III.

### 4.2 RT‐PCR for HO‐1 Expression

**FIGURE 1:**
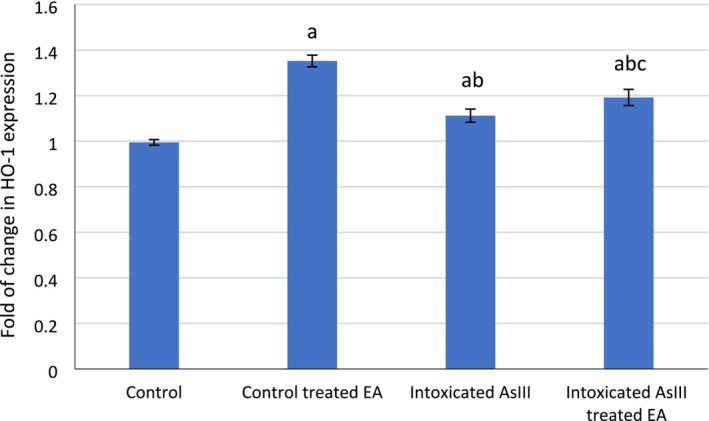
Fold change in HO‐1 expression in quantified and analyzed groups of rats, where HO‐1 was upregulated in EA‐treated, arsenic‐intoxicated group IV compared with group III. Data are presented as mean with error bars representing standard deviation. Statistical analysis was performed using a one‐way ANOVA test. a: means statistical significant difference versus control group, b: means statistical significant difference versus control treated EA group, c: means statistical significant difference versus intoxicated group at *p* < 0.05.

RT‐PCR expression of HO‐1 showed normal levels in Group I. The experiments also indicated that HO‐1 expression levels were elevated in the As^III^‐intoxicated Group III at a dose of 10 mg/kg/b.wt. Meanwhile, the As^III^ and EA 60 mg/kg/b.wt‐treated Group IV showed upregulated protein expression of HO‐1 (Figure 1; Tables 3 and 4). These results suggest that EA supplementation may enhance antioxidant capacity by activating the signaling pathway.

**TABLE 3:** Fold change in HO‐1 expression in different groups of rats.

Control			Control treated EA			Intoxicated As^III^			Intoxicated As^III^ treated EA		
1.003	0.98	1	1.378	1.326	1.352	1.094	1.145	1.096	1.154	1.196	1.225

**TABLE 4:** Statistical analysis was performed by one‐way ANOVA test.

Group	Mean	SD
Control	0.995	0.013
Control treated EA	1.352	0.026
Intoxicated As^III^	1.112	0.029
Intoxicated As^III^ treated EA	1.192	0.035

Comparisons	Significance	*p*
Gp I vs. Gp II	*	< 0.0001
Gp I vs. Gp III	*	0.0031
Gp I vs. Gp IV	*	< 0.0001
Gp II vs. Gp III	*	< 0.0001
Gp II vs. Gp IV	*	0.0004
Gp III vs. Gp IV	*	0.0279

* Means *p* < 0.05, ns means non‐significant.

### 4.3 Immunohistochemical Results (Light Microscopic Detection of VEGF)

**FIGURE 2:**
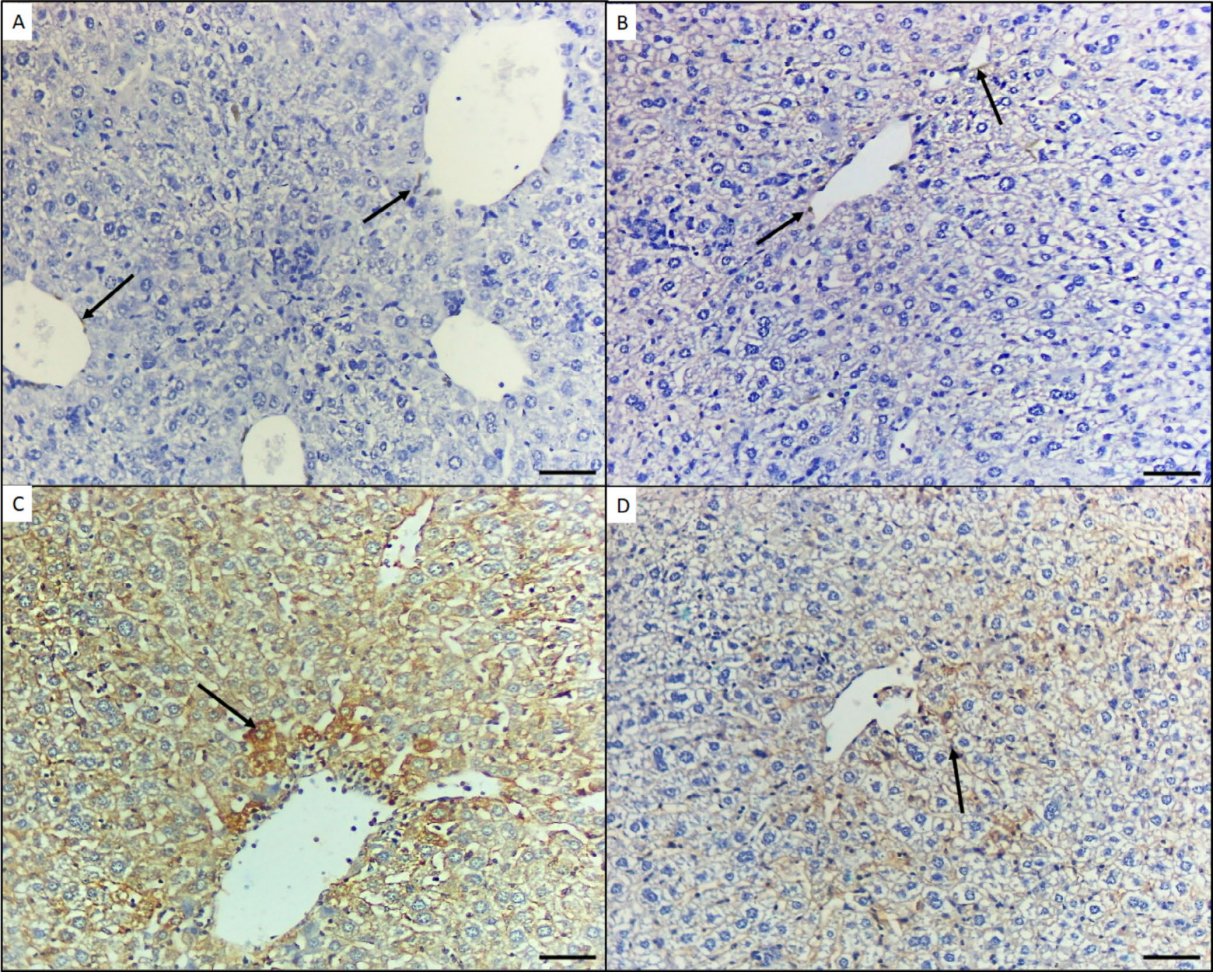
Immunohistochemical staining of vascular endothelial growth factor (VEGF) expression in liver tissues of four groups. (A) Representative minimal VEGF immunohistochemical staining in the normal control group I. (B) Illustrative VEGF staining in group II, showing minimal immunoreactivity. (C) Positive brown staining observed in the intoxicated‐As (10 mg/kg) group III, showing high VEGF immunoreactivity in hepatocytes with increased intensity around the central vein. (D) As (10 mg/kg) + EA (60 mg/kg), group IV, showing weak, moderate, and minimal VEGF staining in hepatocytes. Magnification 200×. Scale bar 50 μm.

Immunohistochemical expression of VEGF‐stained liver sections from Group I and the EA 60 mg/kg/b.wt‐treated rat Group II showed minimal VEGF staining, mainly in the endothelial cells around the central vein. In the As^III^‐intoxicated Group III, the liver showed high VEGF immunoreactivity in hepatic cells, with increased intensity around the central vein. However, the As^III^ and EA 60 mg/kg/b.wt‐treated Group IV showed minimal to mild VEGF immunoreactivity (Figure 2A–D, respectively).

### 4.4 Histopathological Results (Light Microscopic Examination of the H&E‐Stained Tissue Sections)

**FIGURE 3:**
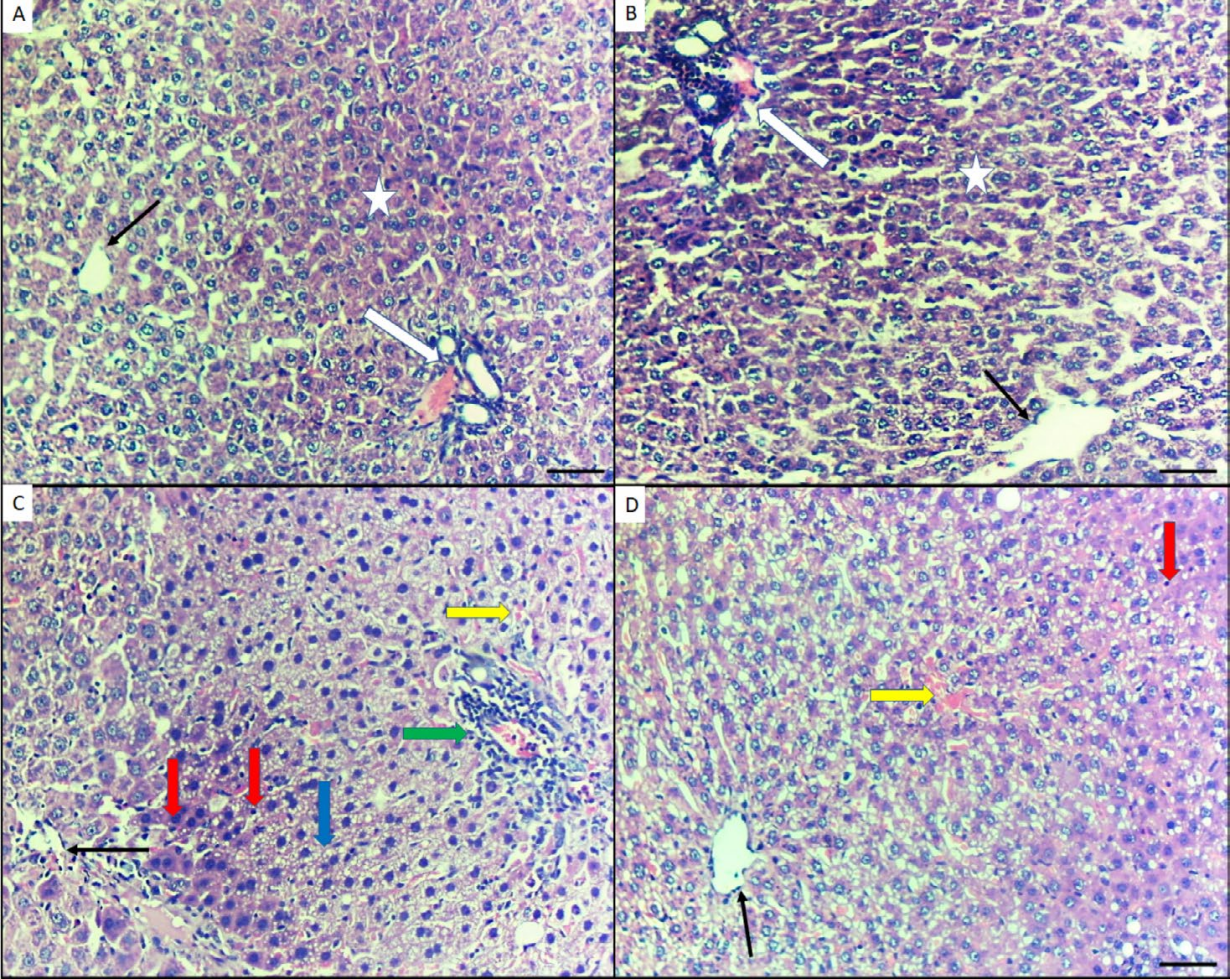
Photomicrograph of histopathological changes in hepatic tissues of all groups (H&E, 200×). (A) Control group I displaying normal histology. (B) EA 60 mg/kg/day treated group II showing normal liver tissue features. (C) As‐intoxicated group III (10 mg/kg/day) showing hepatocyte apoptosis in the form of pyknotic or hyperchromatic nuclei, cytoplasmic vacuolation, and dilated congested sinusoids filled with RBCs. (D) As (10 mg/kg) + EA (60 mg/kg) group showing reduced necrosis and improvement in damaged hepatic tissue, with few signs of pyknotic nuclei. Scale bar 50 μm.

Microscopic examination of H&E‐stained liver sections in control Group I showed normal liver features (Figure 3A). Group II, treated with EA 60 mg/kg/b.wt, also showed normal liver tissue features (Figure 3B). Group III, intoxicated with As^III^ at 10 mg/kg/b.wt, revealed hepatocyte apoptosis in the form of pyknotic or hyperchromatic nuclei, cytoplasmic vacuolation, dilated congested sinusoids filled with RBCs, and inflammation around the portal vein (Figure 3C). However, Group IV, intoxicated with As^III^ and treated with EA, showed only a few signs of hepatocyte apoptosis, such as pyknotic nuclei and dilated congested sinusoids filled with RBCs (Figure 3D).

## 5 Discussion

Our environment is occupied by toxic metals, most of which are produced by mining, industrial processes, and the use of pesticides. One of these is arsenic, which accumulates to dangerous levels in liver tissue, altering the function of many liver enzymes and causing cellular damage (Gaim et al. 2015).

The liver is an important site of ROS production due to its metabolic and detoxification activities. Pathological states can arise from an imbalance between the generation and removal of free radicals. Many biological substances, including lipids, proteins, polysaccharides, and DNA, can be damaged by oxidative stress. Oxidative stress is a major factor in the process of tissue destruction. It occurs when there is an imbalance between the body's capacity to neutralize or repair the harmful effects of ROS and their production (Soliman et al. 2024; Aboubakr et al. 2023).

Enzymes such as AST, ALT, and ALP are predominantly present in liver tissue, and cellular damage due to arsenic exposure leads to the release of these enzymes into the bloodstream. Elevated plasma levels of these enzymes suggest hepatotoxicity (Hatipoglu et al. 2024). These liver enzymes are substantial diagnostic markers for identifying pathophysiological changes resulting from the harmful effects of toxic element exposure and can be used to assess the extent of hepatic damage (Ijaz et al. 2023).

In the present study, As^III^ was administered at 10 mg/kg/b.wt. and resulted in a significant increase (*p* < 0.05) in the levels of ALT, AST, and ALP in Group III compared with Group I. These findings are in agreement with the results of Chen et al. (2023), who reported acute alterations in liver enzymes and observed substantial liver damage with As^III^‐induced hepatotoxicity. These results are also consistent with those of Ramadan et al. (2024), who showed that arsenic administration led to markedly higher levels of ALT and AST in the arsenic‐treated group compared with the control group.

Treatment with EA in Group IV revealed a substantial reduction in serum ALT, AST, and ALP levels. These findings are in agreement with Widyawati et al. (2023) who ascertained that EA reduced liver enzyme profiles by inhibiting cholestasis‐induced liver damage, possibly due to EA's anti‐inflammatory, anti‐fibrotic, and antioxidant properties.

Additionally, in the current study, data showed that As^III^ increased the level of HO‐1 expression in Group III. This is in agreement with Li et al. (2021), who reported that arsenite (As) substantially increased immune tolerance by suppressing the expression of proinflammatory factors, phenotypic molecules, and Th1/Th17‐inducible cytokines in myeloid‐derived dendritic cells stimulated by lipopolysaccharides. Similar findings were reported by Ahmad et al. (2023), who affirmed that As_2_O_3_ considerably induced HO‐1 expression in different PDAC cells through the p38 MAPK signaling pathway. Moreover, our results align with those of Xie et al. (2017), who showed that treatment with As_2_O_3_ enhanced the protein expression of HO‐1.

Nevertheless, in Group IV, results revealed that EA co‐administration with As^III^ further upregulated HO‐1 protein expression compared with Group III (As^III^ only). These findings are in agreement with Zhang et al. (2022) who reported that EA upregulated the mRNA expression of antioxidant enzymes (Nrf2, GPX1, and HO‐1) and demonstrated that both therapeutic and preventive EA treatment were effective in enhancing HO‐1 protein expression.

In the present study, the As^III^‐induced toxicity in rats from Group III exhibited elevated VEGF levels in the serum and high immunoreactivity in hepatocytes, with increased intensity around the central vein. This is in accordance with the findings of Liu et al. (2011) who studied the molecular mechanism of arsenic‐induced angiogenesis and established that arsenic stimulates ROS formation and increases the expression of hypoxia‐inducible factor 1 (HIF‐1) and VEGF.

Conversely, in the Group IV results, the protective impact of EA was evident, with a reduction in VEGF serum levels and mild VEGF immunoreactivity confirmed. These findings are in agreement with Wang et al. (2012) who reported that EA substantially suppressed VEGF‐induced angiogenesis processes such as endothelial cell migration, proliferation, and tube formation. Furthermore, in endothelial cells, it directly inhibited the activity of VEGFR‐2 tyrosine kinase and its downstream signaling pathways, including MAPK and PI3K/Akt.

Similarly, the findings are consistent with those of Kowshik et al. (2014), who demonstrated that EA strongly suppresses PI3K/Akt and MAPK signaling by downregulating PI3K, PDK‐1, p‐Aktser473, mTOR, p‐ERK, and p‐JNK, thereby inhibiting HIF‐1α‐induced VEGF/VEGFR2 signaling in the hamster buccal pouch.

Moreover, the histopathological outcomes showed that hepatocyte injury in the As^III^ toxicity Group III was characterized by pyknotic or hyperchromatic nuclei, cytoplasmic vacuolation, dilated congested sinusoids filled with RBCs, and inflammation around the portal vein. These findings are in agreement with the report by Ijaz et al. (2023) who affirmed that the liver displayed marked necrosis and deterioration in rats administered arsenic. These results are also parallel to those of Ma et al. (2023) who stated that rats exposed to arsenic exhibited an accumulation of total bile acids in both serum and liver, along with abnormalities in liver histology and function.

Concomitant administration of EA and As^III^ in Group IV caused limited signs of hepatocyte injury, observed as pyknotic nuclei and dilated, congested sinusoids filled with RBCs. These findings are consistent with Zhao et al. (2021), who confirmed reduced edema, fibrosis, and inflammatory cell infiltration in groups treated with EA.

Taken together, these results suggest that EA possesses potent anti‐inflammatory and anti‐angiogenic properties. The experimental data support the potential usefulness of EA in controlling hepatic damage and/or injury in experimental models. This action appears to be associated with the modulation of angiogenic factors and the upregulation of HO‐1 expression.

## 6 Conclusion

The present research strongly suggests that EA is a powerful protective agent against liver injury induced by As^III^ exposure. Furthermore, the data from this study revealed that liver damage caused by arsenic intoxication was severe. Although orally supplied EA showed improvement in liver biochemical markers, it also supported hepatic tissue regeneration and regulated protein upregulation. These results imply that EA has exceptional liver damage prevention properties, which could suggest that EA is a good candidate as a preventive agent for liver injury. Subsequent research endeavors should focus on better defining the mechanisms behind EA's protective properties. In addition, further investigations should address the biological and pharmacological properties of EA, which may hold potential for future application in human clinical trials.

## 7 Novelty and Limitations

The findings suggest that HO‐1 upregulation through EA supplementation is a novel approach that could effectively prevent inflammation, reduce angiogenesis, and prevent liver cell death induced by As_2_O_3_. This also offers potential therapeutic approaches for the prevention and management of arsenic‐related toxicity. The limitations of this study include the relatively small sample size and the lack of direct measurements of related proinflammatory cytokine levels and antioxidant activity. Moreover, the suggestion to investigate post‐intoxication treatment with EA after As^III^ exposure should be considered in future studies.

## Author Contributions

T.A., M.F.E.‐R., and E.A.A.A. carried out the experiment. T.A. and M.F.E.‐R. wrote the manuscript. Also, M.F.E.‐R. conceived the original idea. M.F.E.‐R. and E.A.A.A. verified the analytical methods. T.A., M.F.E.‐R., and E.A.A.A. discussed the results and contributed to the final manuscript.

## Ethics Statement

REC/MED/BU‐FM/2024/1.

## Conflicts of Interest

The authors declare no conflicts of interest.

## Data Availability

The corresponding author can provide the data supporting the study's conclusions upon request; at this time, the data are not publicly available but will be made available upon request.
